# Computational challenges and human factors influencing the design and use of clinical research participant eligibility pre-screening tools

**DOI:** 10.1186/1472-6947-12-47

**Published:** 2012-05-30

**Authors:** Taylor R Pressler, Po-Yin Yen, Jing Ding, Jianhua Liu, Peter J Embi, Philip R O Payne

**Affiliations:** 1 Department of Biomedical Informatics, The Ohio State University, Columbus, OH, USA; 2 Information Warehouse, The Ohio State University Medical Center, Columbus, OH, USA

**Keywords:** Clinical trials as topic/Methods, Patient selection, Medical records systems, Computerized, Patients/Classification, User-computer interface

## Abstract

**Background:**

Clinical trials are the primary mechanism for advancing clinical care and evidenced-based practice, yet challenges with the recruitment of participants for such trials are widely recognized as a major barrier to these types of studies. Data warehouses (DW) store large amounts of heterogenous clinical data that can be used to enhance recruitment practices, but multiple challenges exist when using a data warehouse for such activities, due to the manner of collection, management, integration, analysis, and dissemination of the data. A critical step in leveraging the DW for recruitment purposes is being able to match trial eligibility criteria to discrete and semi-structured data types in the data warehouse, though trial eligibility criteria tend to be written without concern for their computability. We present the multi-modal evaluation of a web-based tool that can be used for pre-screening patients for clinical trial eligibility and assess the ability of this tool to be practically used for clinical research pre-screening and recruitment.

**Methods:**

The study used a validation study, usability testing, and a heuristic evaluation to evaluate and characterize the operational characteristics of the software as well as human factors affecting its use.

**Results:**

Clinical trials from the Division of Cardiology and the Department of Family Medicine were used for this multi-modal evaluation, which included a validation study, usability study, and a heuristic evaluation. From the results of the validation study, the software demonstrated a positive predictive value (PPV) of 54.12% and 0.7%, respectively, and a negative predictive value (NPV) of 73.3% and 87.5%, respectively, for two types of clinical trials. Heuristic principles concerning error prevention and documentation were characterized as the major usability issues during the heuristic evaluation.

**Conclusions:**

This software is intended to provide an initial list of eligible patients to a clinical study coordinators, which provides a starting point for further eligibility screening by the coordinator. Because this software has a high “rule in” ability, meaning that it is able to remove patients who are not eligible for the study, the use of an automated tool built to leverage an existing enterprise DW can be beneficial to determining eligibility and facilitating clinical trial recruitment through pre-screening. While the results of this study are promising, further refinement and study of this and related approaches to automated eligibility screening, including comparison to other approaches and stakeholder perceptions, are needed and future studies are planned to address these needs.

## Background

Clinical trials represent a primary mechanism of advancing clinical care and evidence base practice, and as such, are a major area of emphasis for academic health centers (AHC), [1-8]. Challenges with the recruitment of participants for such trials are widely recognized as a major barrier to the timely and efficacious conduct of these types of studies. Ideally, recruitment methods would be able to optimize both the type and number of eligible participants, while also keeping time and monetary expenses at a minimum, [9]. Often, clinical investigators and research staff rely on manual chart reviews to identify potential participants, which is both costly in time and money. If the number of charts to be reviewed by research staff could be reduced through a pre-screening method, there is great potential to facilitate improvements to the clinical trial recruitment process. While many informatics approaches to supporting participant recruitment have been described in the literature, [3,10-14], often labeled as cohort identification or participant recruitment tools, the satisfaction of such information needs remains an open area of research, [6,15,16].

Several types of informatics tools and approaches have been developed and evaluated to address the problem of ineffective clinical trial recruitment. These approaches include techniques that leverage electronic health records (EHRs) to identify potential participants in real-time and trigger alerts at the point-of-care during a physician-patient interaction to facilitate recruitment, [2-7]. Another category of tools involves pre-screening patients for potential trial eligibility prior to a clinical encounter in order to facilitate subsequent contact and manual eligibility assessments by the research team. Both classes of methodologies leverage data from systems such as EHRs and data warehouses (DW); the latter of which is the focus of this paper.

The Ohio State University Medical Center (OSUMC) operates an enterprise data warehouse (DW) termed the Information Warehouse (IW). The structure of the IW is typical of that of most DWs as it houses multiple heterogeneous forms of data, including clinical data collected and stored through EHR systems, as well as billing and administrative data, structured using a modified “snowflake” schema, [17-20]. The IW also includes systems that enable data queries from multiple users including clinicians, researchers, and administrators.

Among the increasingly important uses for DWs like the IW, is the ability to perform clinical trial cohort discovery as well as other data mining and analysis activities intended to facilitate clinical and translational research goals, [21-24]. However, numerous challenges exist when using a DW for activities like cohort discovery, including the manner of collection, management, integration, analysis, and dissemination of the data contained within the DW structure, [6,21-26]. A critical early step in leveraging the DW for recruitment purposes is being able to match trial eligibility criteria to discrete and semi-structured data types (i.e., diagnoses, diagnostic laboratory values, clinical characteristics, procedures, etc.) in the DW. Unfortunately, trial eligibility criteria tend to be written without concern for their computability, thus limiting their ability to be operationalized using rule-engines or database query languages in or in order to satisfy the logical conditions defined by the criteria in an automated manner. Of note, and reflective of this challenge, there is a paucity of literature describing effective approaches to the preceding problem, [27-33].

To address these existing knowledge and performance gaps, we have developed and designed a prototype cohort discovery tool, ASAP (**A**dvanced **S**creening for **A**ctive **P**rotocols), to support eligibility screening against an existing DW (i.e. the OSU IW). Building upon this overall motivation, in the following sections we will briefly describe the design of the ASAP tool, as well as the objectives of the study being reported upon in this manuscript. We present the multi-modal evaluation of the ASAP tool and assess the ability of this tool to be practically used for clinical research pre-screening and recruitment.

### ASAP (advanced screening for active protocols)

**Figure 1:**
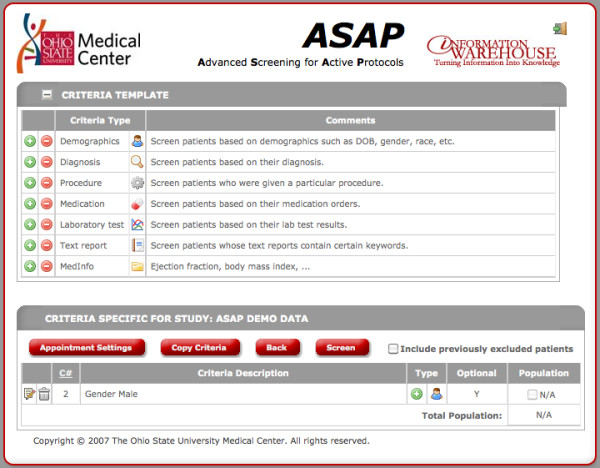
**Screen shot of ASAP tool.** ASAP is an online tool that can be used for prescreening patients for clinical trial eligibility.

Researchers and staff from The Ohio State University Department of Biomedical Informatics (OSU-BMI) and The Ohio State University Medical Center (OSUMC) IW team developed a tool designed to identify and pre-screen patients prior to clinical encounters for the express purpose of clinical trial eligibility assessment. The tool, known as ASAP (**A**dvanced **S**creening for **A**ctive **P**rotocols) is a software solution intended to identify potential participants for clinical trials based on both discrete and semi-structured clinical data in the IW. A major feature of this tool, which differentiates it from other previously reported clinical trials participant screening platforms, is the ability to express and reason upon incomplete or ambiguous data sets, [34,35] Figure 1.

A critical aspect of the design of ASAP was an analysis of prototypical clinical trial eligibility criteria from multiple disease domains in order to identify common query patterns that can be mapped to reoccurring eligibility criteria types. These patterns were used as the basis for developing the ASAP’s query engine, and a full description of this contributing study and design process is provided in our prior reports concerning the described platform, [34]. In its prototypical deployment, ASAP’s user interface model allows research staff to log onto the tool, select the appropriate templates and fill in the data as applicable to the specific clinical trial, screen for patients, and have a listing returned of patients that met the specified criteria. The result set of an executed eligibility checklist includes demographics, encounter scheduling information and details concerning the criteria met by patients who may be eligible for enrollment in the indicated trial. In addition, links are available to directly view source data used during the matching process, such as laboratory values or tagged text reports. Using this to score and stratify potential participants, research staff can heuristically select patients with missing or incomplete data, and perform additional screening interviews to determine their final eligibility status. This type of electronic screening process and heuristic “prioritization” of potential trial participants has previously been shown to improve screening efficiency, [36].

## Results

### Validation

The quantitative results were generated in this study by comparing the number of eligible patients found by the study coordinators using the existing research workflow model (“Gold Standard”) to the number of eligible patients identified by the ASAP tool. These results are grouped by the study type, due to the similarity in the screening criteria, and can be found in Table 1. The sensitivity indicates that ASAP has a sub-optimal ability to rule out ineligible patients, but does demonstrate a functional ability to rule in patients that may be eligible for a clinical trial.

**Table 1:** Validation study results

	**CHF Trials**	**FM Trials**
**Sensitivity**	0.339	0.430
**Specificity**	0.684	0.801
**Positive Predictive Value (PPV)**	54.12%	0.7%
**Negative Predictive Value (NPV)**	73.3%	87.5%

### Heuristic evaluation

**Figure 2:**
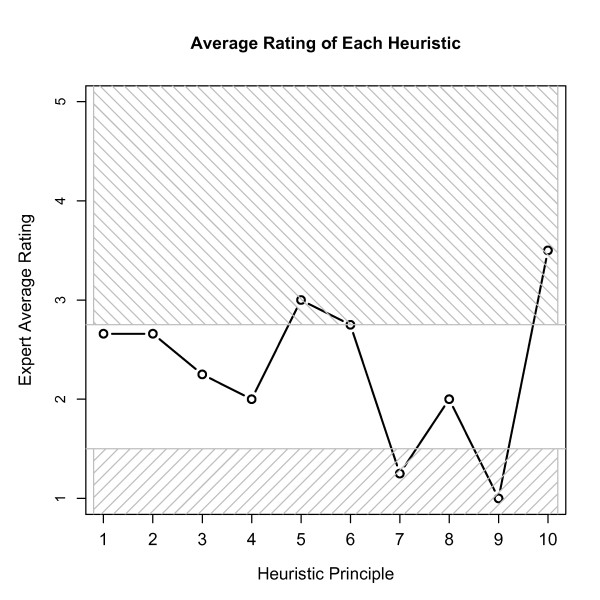
**Average rating of each heuristic principle.** The average expert rating of each heuristic principle from the heuristic evaluation.

The average rating of each heuristic from the heuristic evaluation is displayed in Figure 2 and also given in Table 2 with the classification of the severity of the average rating. The heuristics associated with helping users recognize, diagnose, and recover from errors, error prevention, and documentation had an average rating of at least 2.75 and are thus characterized as major usability problems. Heuristics associated with the visibility of system status, match between system and real world, user control and freedom, consistency and standards, and system flexibility had average scores between 1.5 and 2.75, indicating minor usability problems. The heuristics associated with recognition and recall and minimalist design were characterized as cosmetic usability issues, as the average score was below 1.5.

**Table 2:** Heuristic evaluation results

**Heuristic Principle**	**HCDIM**			**Average Rating**	**Usability Description**
	**%R**	**%T**	**%F**		
1. Visibility of System Status	62.0	34.4	3.4	2.66	Minor
2. Match Between System and Real World	66.6	33.3	0	2.66	Minor
3. User Control and Freedom	4.3	65.2	30.4	2.25	Minor
4. Consistency and Standards	92.1	5.8	1.9	2.0	Minor
5. Help Users Recognize, Diagnose, and Recover From Errors	61.9	38.0	0	3.0	Major
6. Error Prevention	13.3	86.7	0	2.75	Major
7. Recognition Rather Than Recall	95.0	5.0	0	1.25	Cosmetic
8. Flexibility and Ease of Use	0	47.3	43.7	2	Minor
9. Aesthetic and Minimalist Design	100	0	0	1	Cosmetic
10. Help and Documentation	17.3	69.5	13.0	3.5	Major

Results from the heuristic evaluation, including the average rating for each heuristic principle, the proportion of HDCIM items within each heuristic, and the overall rating of the usability for each heuristic.

All items within the heuristic checklist were evaluated for classification in the Human Centered Distributed Information model (HCDIM) and those proportions are also listed in Table 2. The three heuristics that were rated as major usability problems (5, 6, and 10) are primarily comprised of items that captured the elements Task Analysis and Representational Analysis. A small proportion of the items associated with the Help and Documentation heuristic were considered to be a part of Functional Analysis. Based on the HCDIM classifications shown in Table 2, it was expected that the major usability problems encountered by end users would be related to elements within Task and Representation analysis. These HCDIM analysis types indicated that the software either did not match the natural task sequence of the user or the information was not represented in a clear and concise way to the user. The expert comments that are shown in Table 3 indicate that the software did not prevent users from making errors nor did it provide feedback to the user when errors were made. This, in conjunction with the lack of help documentation within the ASAP system, makes it difficult for users to understand the task sequence that is necessary to use the system.

**Table 3:** Select comments from heuristic evaluation

**Heuristic Principle**	**Expert Comments**
5. Help Users Recognize, Diagnose, and Recover From Errors.	“No error messages. I do not know why I can’t retrieve any patients.”“Doesn’t seem to do error checking.”“The system doesn’t help users recognize errors.”“In the Pharmacy Order Code lookup menu, the system displays one record when you enter ‘tylenol’ whereas it shows multiple records when you type in ‘acetaminophen’. Does this mean the user has to use a generic name instead of a brand name for lookup?”“The system doesn’t do error checking or provide feedback.”
6. Error Prevention	“I entered age of 655 by mistake when I meant to enter 65. These kinds of errors should be prevented.”“Can’t navigate between the main menu and the criterion menu.”“I was able to enter an age of 800.”“System doesn’t seem to prevent errors.”
10. Help and Documentation	“No help.”“There is no help function.”“If you don’t know what you are doing, then you are completely stuck!”

The expert comments from the heuristic evaluation regarding the three heuristic principles categorized as 'major usability issues’

### Usability testing

**Figure 3:**
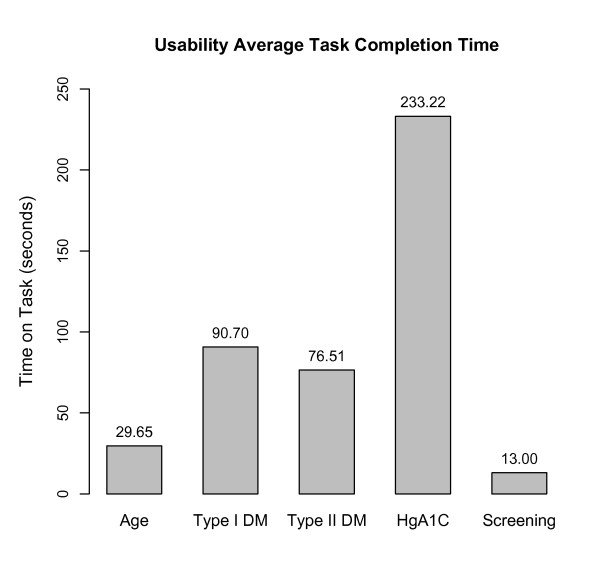
**Usability Average Task Completion Time.** Average time to completion for each task in usability testing.

**Figure 4:**
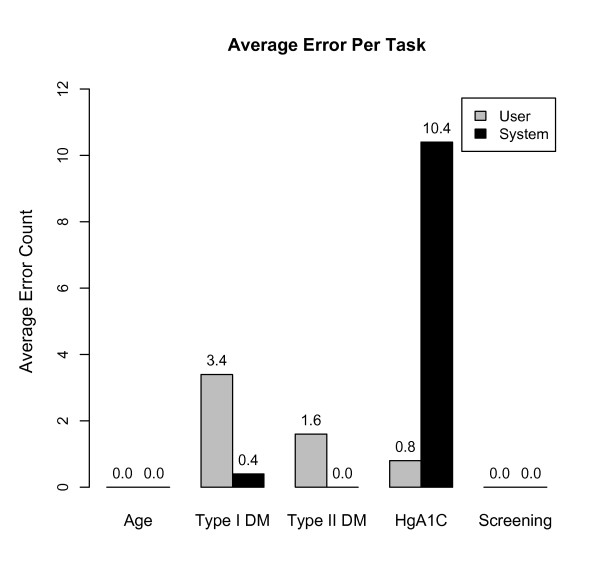
**Average Error Per Task.** Average error frequency for each task in the usability testing.

When asked to perform the set of tasks for the usability testing, the users were generally successful at creating the specified eligibility criteria in under two minutes, including searching for the required codes in order to complete the criteria and entering all pertinent eligibility information. The completion time for each task varied, as show in Figures 3 and 4.

There were several common places where users made errors, which impacted the overall screening process or screening results. These user errors included:

· Did not select appropriate logical reasoning from pull-down menu when selecting multiple ICD-9 codes for a diagnosis (e.g., users did not select “is any of” from the menu and left the choice at the default “is exactly”).

· Did not indicate that the ICD-9 codes should represent a primary diagnosis.

**Figure 5:**
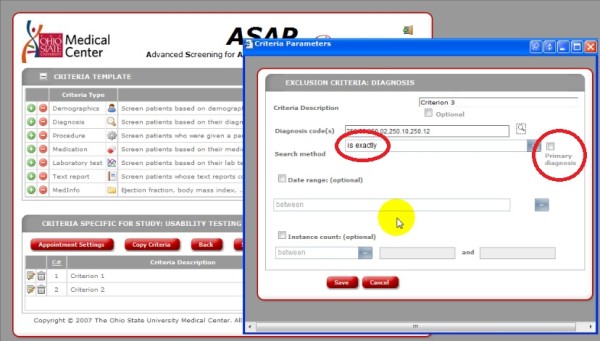
**ASAP Screenshot.** Screen shot from usability testing indicating that the user did not select box for primary diagnosis for correct logical reasoning.

· Did not search through multiple pages of results to select the appropriate laboratory or diagnosis codes Figure 5.

There were several places where users encountered general usability problems, which were considered system errors. System errors were defined as elements of the system that prevented a user from being able to complete a task and included:

· Pull-down menus did not display correctly.

· Calendars did not display correctly.

· Search results varied with synonyms.

· Wording of commands and instructions was unclear.

**Figure 6:**
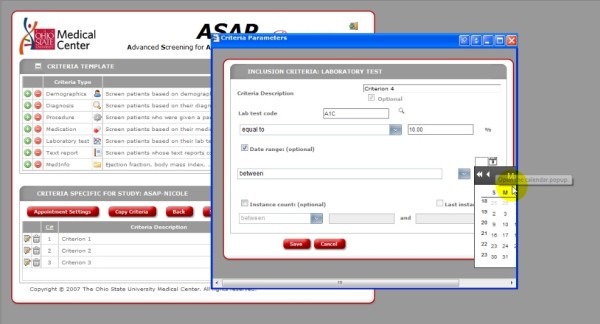
**ASAP Screenshot.** Screenshot from usability testing illustrating the system errors associated with the calendar display.

The calendars had similar issues with display. The user was required to select a date from the calendar to add temporal constraints to the criteria. However, the calendars were not displayed in their entirety and enlarging the size of the window did not satisfy the problem Figure 6.

The search results returned would vary depending on the words entered for the search term (e.g., ‘Tylenol’ versus ‘acetaminophen’ would return different results), which led to inconsistencies in the screening process. Additionally, the wording of some of the logic choices was unclear (‘more than’ versus ‘at least’ for setting limits on laboratory values), which led to confusion and frustration for the user.

The types of issues that the users encountered were consistent with the task and representational analysis definitions of the HCDIM framework and with the predictions from the results of the heuristic evaluation. Issues such as the search term specificity, use of logical constraints in criteria is consistent with Task Analysis and can be approached by further analyzing the information flow and cognitive processes of the users in order to design a new version of the tool that meets the needs of the user. The issues with the display of information in the drop-down menus and calendars is consistent with Representational Analysis and more work can be done to determine the best display of information so that users can interact with the system more effectively.

Despite the problems that users encountered while using the system, the survey results indicate that the users perceived the system to be generally easy to use and potentially useful for pre-screening patients. The average rating for the ease of use of the tool was 3.6 and the average rating for the perceived usefulness of the tool in the participants’ clinical environments was 3.8. Users had positive comments to ASAP, such as “very user friendly and could be learned in a matter of minutes”, the tool “eliminates the time needed to screen each scheduled patient individually”, and “I like how the patient information pulls up with the tabs, making it easy to access the information needed”. They also identified their frustration with the “lack of direction” the tool provides.

## Discussion

ASAP was designed and implemented for use by clinical researchers and their staff members in order to pre-screen patients for clinical trial eligibility prior to a clinical encounter. The tool is not intended to provide a comprehensive eligibility assessment, but rather to screen on some elements of eligibility and to provide electronic access to records so that researchers can further assess the eligibility of a potential participant. Previous studies have indicated that there is a reduction in the time and monetary costs of recruitment as only a subset of charts require manual review after pre-screening, [13,36]. The results of our user survey indicate that there is a need for and an interest in this type of pre-screening tool.

The results of the validation study demonstrate that ASAP has an ability to “rule in” patients that may be eligible for a study based on an initial set of criteria, based on the specificity the NPV values. The tool does not appear to have an equal ability to “rule out” patients that are otherwise ineligible for the study, based on the sensitivity and PPV values. Since this tool is considered a pre-screening tool, as opposed to a decision tool, and meant to provide a preliminary eligibility for study coordinators, ASAP does demonstrate functional ability for screening, based on a previous validation studies of the prediction tool, [37]. The tool presents an initial list of candidates for a clinical trial and it is expected that study coordinators would continue to screen patients for additional study criteria, thus false positives would be removed during that screening mechanism. While further refinement of this tool is necessary to achieve better operational characteristics to characterize it as a replacement to human screening, the results of this validation study are promising for the intended use of ASAP.

It is possible that the structure of the data in the IW may have an impact on the results of the validation study, and thus the operational characteristics of the tool. In order for data to be reliably used and queried from a large, heterogeneous data warehouse structure, it is important that the data are stored in a retrievable format and are stored and shared across multiple programs, [38], and the information can be integrated from multiple sources, [39]. One example of a key criterion that could not be directly and reliably accounted for by the ASAP tool was Ejection Fraction (EF) in the heart failure trials. Though considered one of the most important eligibility criteria, EF values were problematic as they can be reported in multiple types of radiologic reports and often as free text. As a result, ICD-9 codes were used instead as a surrogate for EF in this application of ASAP. These surrogates were clearly not optimal, as the codes lack the granularity necessary to classify patients for physical symptoms and findings. A previous study demonstrated that using ICD-9 codes for screening is not accurate, as the lack of granularity leads to inaccuracy in the identification of diseases, [40]. The use of ICD-9 code surrogates represents one example of key criteria that would certainly create a pool of false- negative results in the ASAP output if not remedied. For tools like ASAP to be useful, they must reliably account for such factors to minimize false negatives. Results of a study by Li et al. suggest that, based on a comparison of NLP and ICD-9 codes used to identify patient eligibility, a combination of structured information, such as ICD-9 codes, and unstructured information, such as clinical narratives, would be useful for identifying eligibility criteria, [41]. The findings from this study ultimately provide evidence that a more consistent approach towards structured data collection in the EHR is needed. By increasing the structure of recorded data, it would allow for a high potential utility in terms of screening patients for trial eligibility, observational studies, and other clinical research data needs.

It is important to note, relative to the preceding limitations concerning the tight coupling of ASAP with the data structures of the OSUWMC IW, that a future direction for the development and evaluation of ASAP can and should focus on its efficacy and utility in more heterogeneous data sharing and re-use environments. For example, the ASAP presentation model and underlying logical controller layers could be easily adapated to consume and reason upon distributed data sets exposed via service oriented architectures such as caGrid [42] or TRIAD [43]. Similarly, these same components could also be coupled with alternative data warehousing platforms, such as the highly-denormalized constructs that underly common, open-source data warehouses such as i2b2 [44]. We intend to pursue the verification and validation of such scenarios as part of future ASAP research and development efforts.

As the quality and granularity of data improves, the usability and quality of data outputs will also improve, [45]. Improvements could include increasing the quality of metadata and including other data description elements such as supporting measurement practice information and possible confounders, [39]. Granularity could also be improved by encoding high priority discrete variables for not just billing purposes, but to classify patients for phenotypic properties. Additionally, improving the sophistication of the database queries could lead to better results. Weng et al. has recently published a study that identified three aspects essential to the construction of database queries for eligibility, [33], which can be used to inform future development of the data structures in the DW and allow for better secondary use of clinical data.

The results of the usability testing and the heuristic evaluation do indicate that there are some areas that should be addressed in order to make this pre-screening tool more efficient and easy to use for pre-screening patients for clinical trial eligibility. One of the biggest efforts this tool should focus on is the creation of a “help” feature and comprehensive user documentation. There was a recognized need for this function by both experts and users. Additional work should also be done to make the labeling of interface components better defined for some of the delimiters when creating the eligibility criteria in the tool. The findings from the usability study are important as, to our knowledge, no literature exists regarding the human factors that predispose or enable end users to adopt a system or tool.

In addition, we recognize that this study does have limitations. One relates to the inability to allow the study coordinators to use the tool in real-time. The weekly reports that were sent to the study coordinators may lead to some patients being missed and may change the results. In addition, this initial study did not include an evaluation of the perceptions of investigators and research staff related to the tool or an examination of how it might be used in other real-world settings as an adjunct to other tools; such studies are planned. We also recognize that the sample size relative to the usability study and the heuristic evaluation is small.

Finally, only four clinical trials were used in this initial evaluation and the findings in this study may not be able to be generalized beyond the domains studied. Ashburn et al. has shown that recruitment is generally better in the elderly population when done through the general practitioner, [46] and would be best suited for an automated alert to the physician. Embi et al. have shown that electronic alerts are able to increase clinical trial recruitment [2]. However, Grundmeir et al. demonstrated that the use of on-site research staff generally lead to recruitment of more subjects for a trial than physician alerts, [47]. The published literature demonstrates both challenges and benefits associated with both types of approaches to trial recruitment, indicating that a combined model should be considered.

While these findings clearly demonstrate that the ASAP tool does not *yet* perform as well as the “Gold Standard” of the human screening workflow, this study does demonstrate that the software does have promise. With further study and development, coupled with improved fidelity and granularity of data within the IW, it may be possible to increase the sensitivity and specificity of the software and to use this tool as one possible means of increasing the clinical trial recruitment rates. In order to accomplish this, future studies will seek to identify the areas where the structure of the data prevents the queries from capturing eligibility information.

## Conclusions

The use of an automated tool built to leverage an existing enterprise DW can be beneficial to determining eligibility and facilitating clinical trial recruitment. While the results of this study are promising, further refinement and study of this and related approaches to automated eligibility screening, including comparison to other approaches and stakeholder perceptions, are needed and future studies are planned to address these needs. This should include further development of the tool to prevent common user errors and issues reported in this study as well as the creation of a ‘help’ feature and comprehensive user documentation. Additionally, further studies are currently examining how the source and the structure of the data within the IW affect the ability of the generalized queries to capture patient information and use it for screening. In summary, ASAP appears to be a promising tool, which can be used to assist in the pre-screening of patients based on an initial set of eligibility criteria.

## Methods

In order to address our objective, our study includes three components: a validation study, heuristic evaluations, and usability testing. This study was reviewed and approved by Ohio State’s Institutional Review Board and is subject to ongoing review.

### Validation study

We conducted an initial evaluation of the ASAP platform using a participatory evaluation design, [48] approach and an assessment of the tool’s sensitivity and specificity. Four clinical study coordinators, two from each group (Chronic Heart Failure (CHF) and Family Medicine (FM)) at OSUMC, were recruited for participation in the study.

Four trials in total were selected for evaluation with each group selecting two trials for the study. A subset of the eligibility criteria for each trial was selected and is summarized below in Table 4. The subset was chosen by the study coordinators with the pre-defined goal of identifying and utilizing general eligibility criteria that would be sufficient in identifying sets of potential trial participants. ASAP was specifically targeted, using these criteria, to screen for patients who were visiting certain clinics and/or physicians as dictated by the clinical trial protocol, thus providing an additional criterion in the identification and pre-screening of potential participants.

**Table 4:** Eligibility criteria

**CHF Trials**		**FM Trials**	
CHF Trial Criteria	CHF Trial Resultant List	FM Trial Criteria	FM Trial Resultant List
Age ≥ 21	*Age ≥ 21*	Age ≥ 18	*Age ≥ 18*
Hospitalization with primary diagnosis of heart failure or cardiomyopathy within the past year.	*Hospitalization within the past 12 months*	Primary diagnosis of Type II Diabetes	*ICD-9 Codes for Type II Diabetes*
Ejection Fraction ≥ 40%&Systolic blood pressure ≥130 mmHg	*ICD-9 Codes for hypertensive heart failure*	Not currently pregnant	*ICD-9 Codes for pregnancy within the past 12 months*
Ejection Fraction ≤ 35%	*ICD-9 Codes for diastolic heart failure*	Hemoglobin A1C values within 7-10%	*7% < HgA1C < 10%*

Clinical trial criteria for both the CHF trials and the FM trials and the resultant list of generalized criteria used for ASAP pre-screening for the validation study.

From this initial subset of appropriate and sufficient criteria, the study investigators and participating study coordinators evaluated the degree to which we could map the elements to those available for querying in the IW using ASAP. Based on that assessment, we refined the eligibility criteria that could be reliably queried by ASAP. The resultant list of criteria for the trials is also shown in Table 4.

Once the criteria selection process was completed, the ASAP tool was put into production using the previously defined criteria. During the four-week period, the study coordinators received an automated screening report generated by ASAP on a weekly basis. The study coordinators would then return weekly data indicating: 1) How many participants on the ASAP screening report were identified through the existing research workflow; 2) how many participants on the ASAP screening report were not identified using the existing research workflow; 3) how many participants were identified through an existing research workflow (manual chart review) and were not included on the ASAP report. The data was then condensed into contingency tables and evaluated using summary statistics and odds ratios.

### Heuristic evaluation

With no training on the system, we used subject matter experts (n = 4) with human-computer interaction backgrounds were asked to perform 4 tasks; including setting criteria based on age, diagnosis, and laboratory values. These experts have relevant research experience and expertise in the areas of human-computer interaction and are published within this field. They were also encouraged to explore other aspects of the system. Each expert completed a heuristic evaluation based on performing a set of typical tasks used to generate the patient eligibility reports. The experts completed the evaluation using the form, [49],which is based on Nielson’s heuristic principles, [50]. This included definitions, sub-questions, and an overall 5-point Likert rating scale for each principle (from 0: no usability problem to 4: usability catastrophe). Also, the Human Centered Distributed Information model (HCDIM) was used to categorize the individual heuristic checklist items and to provide a hypothesis about where usability problems were most likely to occur based on the average heuristic score and the proportional distribution of the analysis types. HCDIM has four analysis types: user, representative, task, and functional, [51]. The user analysis type identifies characteristics of the users and was deemed not appropriate for this study. The following 3 analysis types were utilized in this study:

· Representational analysis is specific to the way information is communicated and the information flow for a given task.

· Task analysis is centered on system functions that must be performed to carry out a task, the flow of information processing between user and system, and the organization and structure of the task.

· Functional analysis is specific to the top-level domain structure of the system and is independent of the implementation, [52].

The individual items within each heuristic checklist were assigned to a category of the HCDIM, [52] by the authors (TRP, PY). A third expert was used to resolve discrepancies in the assignment of checklist items. The 3 selected analysis categories were calculated in relation to each heuristic. The ratings from each completed evaluation were averaged in order to identify issues that could lead to possible usability problems. The results of the heuristic evaluation were used to create hypotheses about the types of issues that would be encountered by the prototypical end users in the usability test, as was executed in the final phase of the study and described below.

### Usability testing

Five clinical study coordinators from Cardiology and Family Medicine were recruited to take part in the usability testing of the software. Each coordinator was asked to perform a series of tasks typical of using the tool for generating eligibility reports, which included creating criteria pertaining to demographic data, criteria selecting appropriate diagnoses and temporal restrictions regarding the date of diagnosis, criteria describing laboratory test values within a certain range, and establishing a date range for when the patient will appear in the clinic (specific task descriptions can be found in Additional file 1). Using a think-aloud protocol, [53], all participants were recorded using the MORAE, [54] software suite and all video recordings were coded to provide an analysis regarding the use of the software tool.

At the end of the usability test, a survey was given to participants (survey can be found in Additional file 1). The survey asked users to rate the system on a Likert scale of 1 to 5 on the ease of use and the perceived usefulness of the tool for the user’s clinical environment. Users were also asked if the tool would be useful for screening patients based on their experience during the usability test.

## Abbreviations

AHC, Academic health centers; ASAP, Advanced Search for Active Protocols; CHF, Chronic heart failure; EHR, Electronic health records; FM, Family Medicine; DW, Data warehouse(s); HCDIM, Human Centered Distributed Information model; IW, Information Warehouse; NPV, Negative predictive value; OSUMC, The Ohio State University Medical Center; PPV, Positive predictive value.

## Competing interests

There are no competing interests to report.

## Authors’ contributions

TP designed and carried out the evaluation study. PY assisted in the design and analysis of the heuristic evaluation. JL and JD designed the software presented in this manuscript and provided the data for the validation study. PE and PP conceived the study, participated in its design and coordination, and helped to revise the final manuscript. All authors have read and approved the final manuscript.

## Pre-publication history

The pre-publication history for this paper can be accessed here:

http://www.biomedcentral.com/1472-6947/12/47/prepub

## Supplementary Material

Additional file 1 **Supplemental Material.** The task list and survey presented to the users during the usability testing portion of the study. Click here for file
