# Supplementary material for: Computational challenges and human factors influencing the design and use of clinical research participant eligibility pre-screening tools
Source: BMC Med Inform Decis Mak. 2012 May 30;12:47. doi: 10.1186/1472-6947-12-47 (PMC3407791; doi:10.1186/1472-6947-12-47)
Supplement: Additional file 1 — Supplemental Material. The task list and survey presented to the users during the usability testing portion of the study. [file 1472-6947-12-47-S1.doc]

**SUPPLEMENTAL MATERIAL**

**Usability Task List:**

Please set up the following criteria in ASAP for the specified protocol.

1. Inclusion Criteria: age 35-80
2. Inclusion Criteria: diagnosis of general diabetes or Type I diabetes.
3. Exclusion Criteria: diagnosis of Type II diabetes.
4. Optional Inclusion Criteria: Hemoglobin A1C value of >10% within the past 3 years.

**End User Survey:**

1. How many patients were screened as eligible for this study protocol?
2. Did any patients match for all of the available criteria?
3. Which of the optional criteria (if any) did not have any patient matches?
4. Choose a patient from the available list.  Can you locate the patient's next appointment?  If so, when is the next visit for this patient and with whom?
5. How easy to use was the ASAP tool? (Likert scale 1-5: 1=Very difficult, 5=Very Easy)
6. Based on today's session, do you feel that ASAP could be useful to screen patients for clinical studies? (Yes/No with comment box)
7. How useful do you think ASAP would be in your clinical research environment? (Likert scale 1-5: 1=Not useful at all, 5=Very Useful)
8. What do you like most about the ASAP tool based on today's experience?
9. What do you like least about the ASAP tool based on today's experience?
10. Any other comments that you would like to share with the researchers and/or creators of ASAP based on your experience today?
